# Protective Effect of *Urtica dioica* Extract against Oxidative Stress in Human Skin Fibroblasts

**DOI:** 10.3390/life13112182

**Published:** 2023-11-09

**Authors:** Agnieszka Skalska-Kamińska, Weronika Wójciak, Magdalena Żuk, Roman Paduch, Magdalena Wójciak

**Affiliations:** 1 Department of Analytical Chemistry, Medical University of Lublin, Chodźki 4a, 20-093 Lublin, Poland; agnieszka.skalska-kaminska@umlub.pl (A.S.-K.); weronikawojciak01@gmail.com (W.W.); magdalena.zu25@gmail.com (M.Ż.); 2 Department of Virology and Immunology, Institute of Biological Sciences, Faculty of Biology and Biotechnology, Maria Curie-Skłodowska University, 19 Akademicka Street, 20-033 Lublin, Poland; roman.paduch@mail.umcs.pl; 3 Department of General and Pediatric Ophthalmology, Medical University of Lublin, Chmielna 1, 20-079 Lublin, Poland

**Keywords:** nettle, fibroblasts, antioxidants, chromatographic analysis, oxidative stress

## Abstract

*Urtica dioica* is a species with well-established significance in folk medicine in many countries. It was utilized to support the treatment of arthritis, allergies, and urinary tract disorders; however, the substantial presence of antioxidants suggests that nettle extract could also have a positive impact on the skin. The objective of this study was to assess the impact of nettle extract on human skin fibroblasts subjected to oxidative stress. Various solvents were tested to prepare an extract rich in polyphenolic compounds with high antioxidant potential. The chemical composition was determined using ultra-high-performance liquid chromatography with mass spectrometry (UPLC-DAD-MS). H_2_O_2_ treatment was used to induce oxidative stress and cell viability, and the metabolism was evaluated through NR and MTT assays. Our study demonstrated that extraction with 80% ethanol, followed by the drying and re-dissolving of the extract in pure water, was more efficient than direct extraction with water. This yielded an extract rich in polyphenolic compounds, with chlorogenic acid and caffeoylmalic acid as the predominant compounds, averaging 64.9 and 114.4 µg/mL, respectively. The extract exhibited antioxidant properties in the DPPH and ABTS assays. Furthermore, it did not exhibit cytotoxicity and did not negatively affect cell metabolism. In addition, it effectively reduced ROS in the H_2_O_2_-stimulated cells, and at the highest concentration tested, the ROS levels returned to those of the untreated control. The extract also protected against H_2_O_2_-induced cytotoxicity. The cell viability was maintained at the level of the untreated control when the cells were pretreated with the extract before H_2_O_2_ exposure. These findings indicate that *U. dioica* extract is a valuable and safe additive in skincare products.

## 1. Introduction

*Urtica dioica*, which is more commonly known as the stinging nettle, common nettle, or burn nettle, is a perennial flowering plant that belongs to the Urticaceae family. Its most recognized feature is the distinctive stinging hairs on its leaves and stems, which release an irritating agent causing a mild to moderate skin reaction. Its roots, stems, and leaves have been used for various medicinal and culinary purposes across cultures for centuries. The plant is rich in nutrients, including vitamins such as B6, B2, A, and K, as well as minerals like magnesium, manganese, and calcium [1]. Tannins, volatile substances, fatty acids, polysaccharides, sterols, terpenes, and polyphenols, such as phenolic acids and flavonoids, are also among chemical components of this plant [2,3,4].

*U. dioica* is a species of significant importance in folk medicine worldwide, including that of European, Asian, North African, and North American countries. The plant was used in various forms, including extracts, decoctions, juice, and infusions, to support the treatment of arthritis, rheumatism, allergies, and urinary tract disorders. It was also applied externally as an ointment to alleviate burns, sunburns, and insect bites [5,6,7].

Contemporary scientific studies have confirmed the efficacy of nettle preparations, justifying its traditional usage. Researchers have found that it exhibits anti-inflammatory, antioxidant, antibacterial, and analgesic activities [1,8,9]. Furthermore, nettle has demonstrated anti-proliferative, anti-hypertensive, and anti-diabetic effects [10,11].

The substantial presence of antioxidants also suggests that nettle extract could be a valuable additive to skincare products. It is well known that oxidative stress, induced by the presence of reactive oxygen species (ROS), initiates unfavorable processes in the skin. The ROS impact the skin micro-environment, leading to extracellular matrix (ECM) degradation [12]. They further contribute to collagen fragmentation and the disorganization of collagen fibers, resulting in structural and functional alterations within the skin. Additionally, ROS can cause cell damage, leading to an increased production of pro-inflammatory cytokines. All these processes accelerate skin aging and contribute to the development of chronic inflammation [13]. Therefore, the ability to reduce free radicals is a highly desirable feature of various cosmetics as it could possibly slow down the process of skin aging [14].

Despite nettle’s well-established position in ethnopharmacology and the numerous mentions of its effectiveness in addressing dermatological problems, there is still an insufficient number of scientific studies verifying its impact on skin. To date, it has been established that nettle extracts possess significant wound-healing properties that can accelerate the repair of damaged skin [15,16,17]. Furthermore, *U. dioica* demonstrated antiaging properties linked with the inhibition of elastase and collagenase [18] and showed anti-androgenic activity in HaCaT cells; therefore, it may prevent the development androgenic skin diseases [19]. Considering the topical application of nettle extract as a cosmetic additive, the study aimed to assess the antioxidant and protective effects of *U. dioica* on human skin fibroblasts. H_2_O_2_ stimulation was used in the investigation as a model of oxidative stress.

## 2. Materials and Methods

### 2.1. Sample Preparation

*Urtica dioica* plants were collected in August 2023 from the area of the Botanical Garden in Lublin (51°16′ N, 22°30′ E). The leaves were washed under running water and freeze-dried (0.001 mbar) for 48 h using a Christ Alpha 2–4 LDplus dryer (Martin Christ Gefriertrocknungsanlagen, GmbH, Osterode am Harz, Germany). The freeze-dried leaves were pulverized and accurately weighed [20]. Two procedures of extract preparation were carried out: (i) the extracts were prepared directly from 20 mg of freeze-dried leaves using 2 mL of 10% polypropylene glycol in water (DG), 5% ethanol in water (DE), and pure water (DW); (ii) 20 mg of freeze-dried leaves was extracted using 2 mL of 80% methanol (MDE); the solvent was evaporated to dryness, and the residues were re-dissolved using 2 mL of 10% polypropylene glycol in water (DRG), 5% ethanol in water (DRE), and pure water (DRW). The extraction was assisted by ultrasound and lasted 15 min.

For phytochemical profiling, 100 mg of the freeze-dried leaves was extracted using 80% methanol followed by 100% methanol. The extracts were centrifuged, and the supernatants were combined, concentrated to a final volume of 5 mL, and filtered.

### 2.2. Chromatographic Analysis

The samples were analyzed with the use of an ultra-high performance liquid chromatograph (UHPLC) Infinity Series II with a DAD and MS detector (Agilent Technologies, Santa Clara, CA, USA). The separation conditions were as previously described [21]. A Titan column (10 cm length, 2.1 mm i.d., 1.9 µm particle size) (Supelco, Sigma-Aldrich, Burlington, MA, USA) and a mobile phase composed of water (A) and acetonitrile (B), both acidified with 0.05% formic acid, were used to achieve the separation. All the solvents were MS grade (Sigma-Aldrich). The elution conditions were: 0–8 min from 98% A to 93% A; 8–15 min from 93% A to 88% A; 15–29 min from 88% A to 85% A; 29–40 min from 85% A to 80% A; and 40–60 min from 80% A to 65% A. The temperature and flow rate were set at 30 °C and 0.2 mL/min, respectively. Electrospray ionization in negative mode was used in the MS analysis. Ions were collected in the range from 100 to 1000 m/z. The drying gas temperature and flow rate were 325 °C and 8 L min^−1^, respectively. The nebulizer pressure was 30 psi, and the capillary, skimmer, and fragmentator voltages were 3500 V, 65 V, and 220 V, respectively. Quantification was carried out using an external calibration method. Solutions of standards (Sigma-Aldrich) at a concentration of 1 mg/mL were used to prepare working solutions in appropriate calibration ranges. The calibration curves were generated by plotting peak areas vs. concentrations. The calibration data are summarized in Table S1.

### 2.3. Antioxidant Assay

The 2,2-diphenyl-1-picrylhydrazyl (DPPH) test and ABTS scavenging assay were carried out according to a previously published procedure [22]. The samples were mixed with DPPH solution (Merck KGaA, Darmstadt, Germany) at a concentration of 4 mM. The absorbance was measured using a UV-VIS Filter Max 5 spectrophotometer (Thermo Fisher Scientific, Waltham, MA, USA) at λ = 517 nm. The distilled water with DPPH solution was used as a control. For the ABTS assay, a mixture of 7 mM ABTS solution (Merck KGaA, Darmstadt, Germany) with 2.4 mM potassium persulfate at a 1:1 ratio was prepared and left at room temperature for 14 h. Afterwards, the solution was adjusted to an absorbance of 1.0 ± 0.04 at a wavelength of 734 nm. The ABTS solution was mixed with the extracts, and the absorbance was measured at a wavelength of λ = 734 nm.

### 2.4. Cell Assays

Human skin fibroblasts BJ CRL-2522™ (American Type Culture Collection, Manassas, VA, USA) were cultured following the manufacturer’s recommendations. After reaching confluence and the removal of the culture medium, the cells were washed with sterile phosphate-buffered saline (PBS) (Gibco). Subsequently, they were trypsinized using 0.25% Trypsin/EDTA (Gibco) and then re-suspended in fresh medium. For the cytotoxic tests, the cells were seeded at a density of 1 × 10^5^ cells/mL in the wells of 96-well plates. After 24 h of incubation at 37 °C, the cells were exposed to the extract. In order to assess the protective effect and reactive oxygen species (ROS) scavenging activity of the extract, the cells were pretreated with the extract for 60 min. Following the pretreatment, H_2_O_2_ (250 µM) was introduced to the medium to induce oxidative stress [23].

#### 2.4.1. Cell Viability Assay

MTT Assay: 3-(4,5-dimethylthiazole-2-yl)-2,5-diphenyltetrazolium bromide solution (MTT) (Sigma) at a concentration of 5 mg/mL in PBS (25 μL/well) was added to the cells cultured in 100 μL of the medium. After 3 h of incubation at 37 °C, 100 μL of 10% sodium dodecyl sulfate (SDS) in a 0.01 M HCl solution was added, and the sample was solubilized overnight. The absorbance was measured at λ = 570 nm using an E-max Microplate Reader (Molecular Devices Corporation, Menlo Park, CA, USA) [20].

Neutral Red Uptake Assay: After the incubation of the cells with the extract, the culture medium was discarded and neutral red dye (NR) at a concentration of 40 μg/mL in the culture medium was added to the cells (100 μL/well). After a 2 h incubation period at 37 °C, the NR solution was aspirated, and 200 µL of a solution containing 0.5% formalin in 1% CaCl_2_ was added. After cell fixation for 3 min, the formalin solution was discarded; this was followed by the addition of 150 µL of a decolorizing buffer composed of 1% glacial acetic acid in 50% ethanol. The plates were shaken for 10 min, and the absorbance was measured at λ = 540 nm using an E-max Microplate Reader (Molecular Devices Corporation) [20].

#### 2.4.2. Analysis of Intracellular Levels of Reactive Oxygen Species (ROS)

After adding the fluorogenic H_2_DCFDA, the cells were incubated in the dark for 45 min and then the level of 2′,7′-dichlorofluorescein (DCF) fluorescence was measured. The excitation and the emission wavelengths were λ = 485 nm and λ = 530 nm, respectively. The measurements (n = 3) were carried out using a microplate reader (FilterMax F5, Thermo Fisher Scientific, Waltham, MA, USA). Ascorbic acid (AA) at concentration of 50 µg/mL was taken as a positive control.

#### 2.4.3. May–Grünwald–Giemsa Staining

The cells were incubated with 1 mL of culture medium containing the tested compounds. After 24 h of incubation (37 °C in humidified 5% CO2/95% air), the medium was removed, the cells were rinsed with culture medium and stained with May–Grünwald (MG) stain for 5 min, followed by staining for another 5 min in MG mixed with water 1:1, *v*/*v*. The MG was removed, and a Giemsa reagent (diluted 1:20 in water) was added to the cells. After 15 min incubation at room temperature, the cells were rinsed with water, dried, and observed using an Olympus microscope (BX51, Olympus, Tokyo, Japan).

### 2.5. Statistical Analysis

All the analyses were replicated three times. The data were presented as the mean ± standard deviation (SD). The data were analyzed utilizing Statistica ver. 13.3 software accessed on 8 February 2021 (https://www.statsoft.pl/statistica_13). One-way ANOVA, followed by Dunnett’s post hoc test was carried out.

## 3. Results

### 3.1. UPLC-MS Profiling of Plant Extract

**Figure 1:**
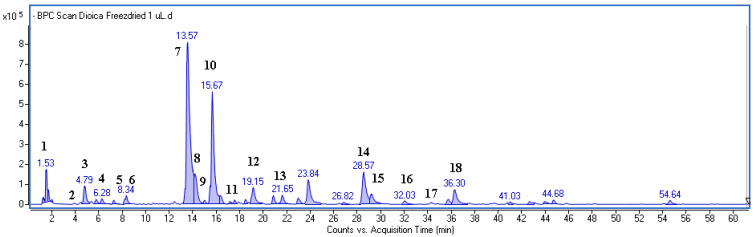
BPC chromatogram of *Urtica dioica* extract obtained in negative ionization mode. The names of identified compounds are given in Table 1.

The identification of the plant phenolic components was based on comparing the UV-Vis spectra, MS data, and retention times with the standards. When the standards were not available, the identification relied on the analysis of the fragmentation patterns of the compounds and the existing literature data [24]. An example of a base peak chromatogram (BPC) is shown in Figure 1. A DAD chromatogram, along with the UV-Vis spectra of the main components, is provided in the Supplementary Material (Figure S1 and Table S2).

Table 1 summarizes the retention times, the mass data, and the discrepancies between the obtained mass values and the theoretical mass values for the estimated formulas. The chemical formulas of the compound were established using the MassHunter formula calculator.

The predominant constituents of the analyzed extract were caffeic acid (CA) derivatives, identified by a common fragment ion with m/z-H = 179, which corresponds to caffeic acid. Among them, caffeoylmalic acid and chlorogenic acid (esters of CA and quinic acid) were the most abundant, and their amounts were 6.38 and 3.58 mg/g of freeze-dried leaves, respectively. Furthermore, cryptochlorogenic, caffeic, ferulic, and coumaroylquinic acids were detected in the sample, with average contents of 0.35, 0.13, 0.11, and 0.50 mg/g, respectively. Some flavonoids belonging to quercetin glycosides (common fragment ion m/z-H = 300) were also found in the extract, including rutin (quercetin rutinoside) and isoquercetin (quercetin glucoside), with quantities of 0.74 and 0.25 mg/g of freeze-dried leaves, respectively. Quinic acid (cyclohexanecarboxylic acid) was determined to be present at an amount of 0.21 mg/g.

### 3.2. Quantitative Analysis of Main Polyphenolic Components of the Extracts

**Figure 2:**
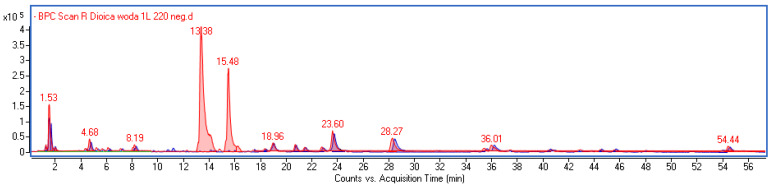
Overlapping BPC chromatograms of extracts obtained through direct extraction with water (blue line) and through the re-dissolution of methanol/water extract in water (red line).

The extracts were prepared either by directly extracting freeze-dried material (D) or by re-dissolving the methanol/water extract that had been evaporated to dryness (DR). All the solvents used, including 10% polypropylene glycol in water (G), 5% ethanol in water (E), and pure water (W), are approved for direct application on the skin. The extracts were analyzed using UPLC-MS-DAD, and both the qualitative and quantitative profiles of the extracts were compared. There were significant differences between the extracts obtained through the direct extraction of freeze-dried leaves and those prepared by dissolving the dried methanol extract (Figure 2). Notably, the derivatives of caffeic acid were not directly extracted using the selected solvent mixtures; however, they were found at high concentrations in the extracts obtained by re-dissolving the residues from the methanol/water extract. Conversely, the extracting mixture did not significantly affect the qualitative profile, and the chromatograms of extracts prepared in the same way appeared similar (Figures S2 and S3). The quantification results for the identified components in the samples, expressed as µg/mL, are presented in Table 2.

### 3.3. Antioxidant Activity

**Figure 3:**
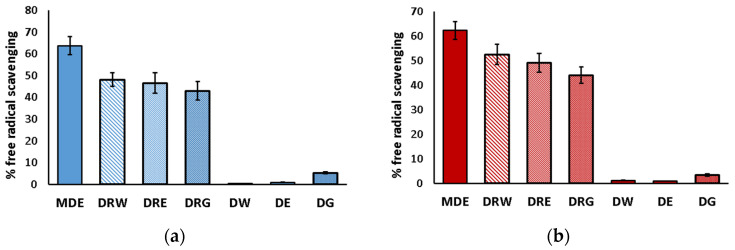
Free radical scavenge activity of *Urtica dioica* extracts determined by the DPPH (**a**) and ABTS (**b**) assay. The extracts were obtained by extraction of freeze-dried leaves with 80% methanol (MDE); next, the extract was evaporated to dryness and re-dissolved in distilled water (DRW), 5% ethanol (DRE), and 10% propylene glycol (DRG). DW—extract obtained using water, DE—extract obtained using 5% ethanol, DG—extract obtained using 10% propylene glycol. Error bars mean the standard deviation.

The DPPH and ABTS methods were used to assess the antioxidant activity of the extracts, and their antioxidant potential was compared based on their ability to scavenge free radicals, which was expressed as a percentage (Figure 3). Generally, the free radical scavenging effect was highly correlated with the polyphenol content, and, as a result, the extracts obtained through direct extraction exhibited negligible potential. Interestingly, a decrease in antioxidant potential was observed in the extracts obtained by dissolving the evaporated ethanol/water extract when compared with the primary extract (MDE), indicating that some antioxidants were not dissolved in the tested solvents.

### 3.4. Cells Based Assays

Based on the results of the quantitative analysis of the polyphenolic compounds and the potential free radical scavenging activity, the DRW extract was selected for the cell assays. It contained the highest amount of phenolic acids, and it also had the highest ROS scavenging activity. Cell viability was evaluated based on measurements of the cellular metabolic activity (MTT assay) and the integrity of the cell membranes (NR assay).

#### 3.4.1. Cytotoxicity Tests

**Figure 4:**
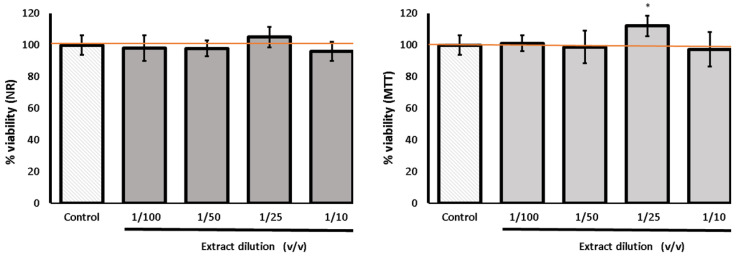
Effect of the different dilutions of *Urtica dioica* extract on cell viability determined by the NR and MTT assay and calculated as a % of control (set as 100%). Control cells were cultured in medium with 0.5% DMSO addition. The extract was obtained by extraction of freeze-dried leaves with 80% methanol; next, the extract was evaporated to dryness, re-dissolved in distilled water (DRW), and then diluted in PBS. The data are means (n = 3) ± SD. One-way ANOVA followed by Dunnett’s post hoc test; * indicates statistically significant difference between samples and control at *p* < 0.05. Error bars mean the standard deviation.

Cytotoxic analysis conducted after the incubation of the cells with various dilutions of the tested extract revealed no adverse effects on cell metabolism or cell membrane integrity (Figure 4). The values remained at the control level, and a slight proliferative effect was even observed in the MTT assay for the 1/25 *v*/*v* dilution. These data provide evidence that the extract is safe for fibroblast cells.

#### 3.4.2. Antioxidant and Protective Activity of the Extract

**Figure 5:**
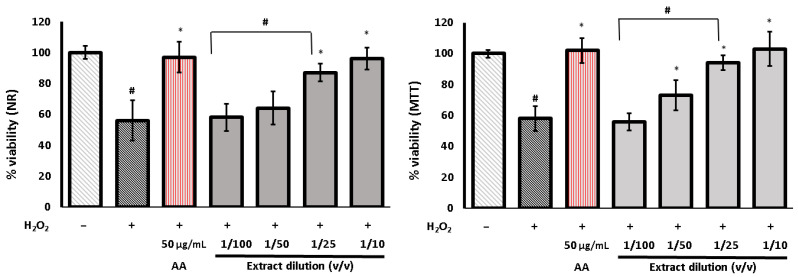
Effect of the different dilutions of *U. dioica* extract on cell viability determined by the NR and MTT assay and expressed as a % of control (set as 100%) in H_2_O_2_ stimulated human skin fibroblasts. Control cells were cultured in medium with 0.5% DMSO addition. The extract was obtained by extraction of freeze-dried leaves with 80% methanol; next, the extract was evaporated to dryness and re-dissolved in distilled water (DRW) and diluted with PBS. Cells were pretreated with the extract prior to the H_2_O_2_ exposure. The data are means (n = 3) ± SD. * indicates statistically significant difference vs. H_2_O_2_-stimulated cells and # indicates statistically significant difference vs. H_2_O_2_-stimulated cells pretreated with ascorbic acid (AA). One-way ANOVA followed by Dunnett’s post hoc test was used (*p* < 0.05). Error bars mean the standard deviation.

**Figure 6:**
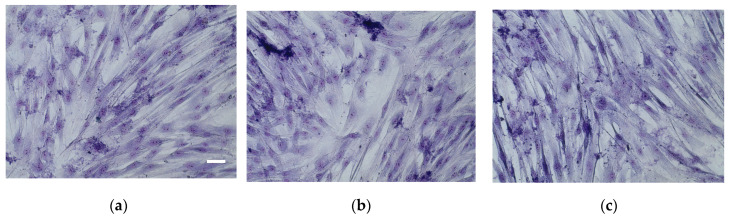
Human fibroblasts cells stained by May–Grünwald–Giemsa dyes. (**a**)—control, (**b**)—cells treated with H_2_O_2_, (**c**)—cells pretreated with *U. dioica* extract at dilution 1/25 *v*/*v* prior to the H_2_O_2_ exposure. Bar = 20 µm.

Figure 5 and Figure 6 depict the effects of the DRW extract on the fibroblasts subjected to induced oxidative stress. Under experimental conditions, a significant reduction in cell viability was observed in the cells treated with H_2_O_2_; this reduction exceeded 40% compared to the untreated control. However, when the cells were preincubated with the extract before the addition of H_2_O_2_, the cell viability was significantly higher than that of H_2_O_2_-stimulated cells. This effect was concentration-dependent, and at the lowest dilution, cell viability was maintained at the level of the untreated control values. This suggests that the addition of the extract reduces the adverse cellular effects of H_2_O_2_ and acts protectively under oxidative stress conditions.

**Figure 7:**
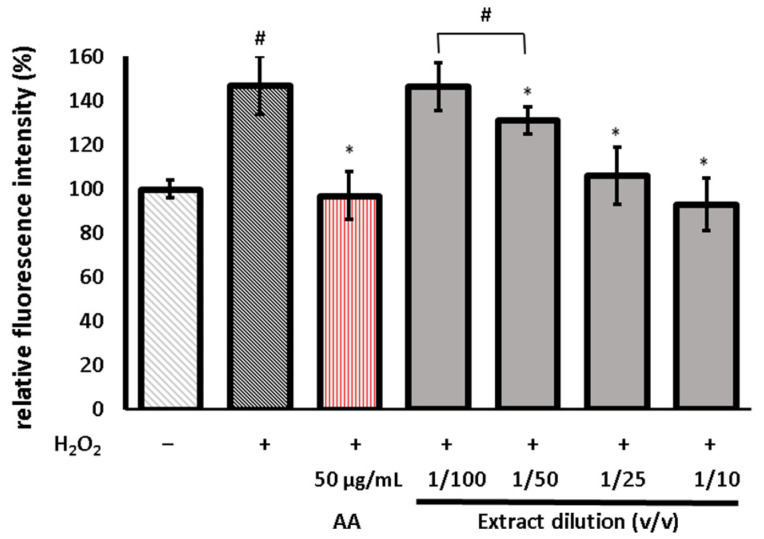
Relative fluorescence of 2′,7′-dichlorodihydrofluorescein (DCF) in human skin fibroblasts, calculated as a percentage in comparison to untreated control cells. Cells were pretreated with different dilutions of the extract or ascorbic acid (AA) prior to the H_2_O_2_ exposure. The extract was obtained by extraction of *U. dioica* with 80% methanol; next, the extract was evaporated to dryness and re-dissolved in distilled water (DRW) and diluted with PBS. The data are means ± SD (n = 3). * indicates a statistically significant difference vs. H_2_O_2_-stimulated cells and # indicates statistically significant difference vs. H_2_O_2_-stimulated cells pretreated with AA. One-way ANOVA followed by Dunnett’s post hoc test was used (*p* < 0.05). Error bars mean the standard deviation.

Further assays using H_2_DCFDA were conducted to assess whether the protective effect on the cells was linked to an impact on the redox imbalance in the H_2_O_2_-induced cells. The test protocol is based on the diffusion of a fluorogenic dye into the cell, where it is subsequently deacetylated by cellular esterases. Later, it undergoes oxidation by ROS, and fluorescent 2′,7′-dichlorofluorescein (DCF) is created. As observed in Figure 7, the H_2_O_2_ treatment significantly increased the ROS levels. The preincubation of the cells with the extract reduced the ROS production in a concentration-dependent manner. At the highest concentration of the extract, the ROS levels returned to those of the untreated control.

## 4. Discussion

Plant extracts are a rich reservoir of valuable phytochemicals with various biological properties. Among them, plant polyphenols have gained increasing attention because they possess multidirectional activity and are known to support human health and protect against the development of different disorders [25,26]. Their effectiveness is mostly related to antioxidant activity and prevention against oxidative stress. Therefore, plants with high antioxidant content are appreciated as additives in skincare products, given the well-established role of oxidative stress in the development of skin-related diseases and skin aging [27].

Our work demonstrated that *U. dioica* is rich in polyphenolic compounds, which are known for their ability to scavenge free radicals. In general, the phytochemical profile and the results of the quantitative analysis were consistent with the majority of the literature data, and the values obtained were within the reported range. According to previously published papers, caffeoylmalic acid (CMa) and caffeoylquinic acid (CQa) were identified as the predominant constituents of *U. dioica* [24,28,29,30,31]; however, their content varied depending on the time of harvesting, region, or processing method. The type of extraction solvent and the method of sample preparation could also significantly affect the results due to their impact on extraction efficacy [3,24,28,32].

In a three-year study, Grevsen et al. found that the amounts ranged from 4.6 to 21.8 mg/g for CMa and from 2.5 to 18.3 mg/g for CQa, depending on the time of collection and the application of nitrogen fertilization [28]. In turn, Garcìa et al., who investigated the impact of different methods for drying of plant material on the content of metabolites, found that the content of CMa ranged from 3.0 to 13.7 mg/g, and the content of total CQas ranged from 10.8 to 20.7 mg/g, depending on the method used [32]. As for the other constituents of the plant, the reported values were as follows: 0.002–9.8 mg/g of rutin, 0.12–4.8 mg/g of isoquercetin, 0.06–0.1 mg/g of ferulic acid, 0.16–0.40 mg/g of cafferic acid, and 0.18–0.25 mg/g of quinic acid [28,32]. For comparison, in the present study, we found 6.4 mg/g of CMa, 3.9 mg/g of total CQas, 0.74 mg/g of rutin, 0.25 mg/g of isoquercetin, 0.11 mg/g of ferulic acid, 0.13 mg/g of cafferic acid, and 0.21 mg/g of quinic acid.

In our study, the antioxidant properties of different *U. dioica* extracts were evaluated in the context of potential cosmetic applications. The composition of the extraction mixture was carefully selected to avoid the solvents prohibited in cosmetology and to stay within permitted organic solvent concentration limits. The choice of extraction solvent is of great importance as it dictates the composition of the final product and, consequently, its biological activity [33]. Water is the safest and most environmentally friendly solvent. However, our study revealed that it has limited effectiveness in isolating caffeic acid derivatives, even when supplemented with low amounts of the organic solvents permitted in cosmetics, like ethanol and polypropylene glycol. According to the literature data, polyphenols are more effectively extracted using mixtures with methanol/ethanol concentrations in the range of 60–90%. Such mixtures exhibit increased solubility of the compounds and higher mass transfer velocities, as well as reduced viscosity and surface tension, which enhances the penetration into plant material [33,34]. Therefore, the two-step process involving initial extraction using 80% methanol, followed by the evaporation of the harmful solvent and the subsequent dissolution of the residues in the mixtures permitted for skin application, was utilized in our work. This solution ensured a polyphenol-rich extract, as confirmed through chromatographic analysis. The presence of these metabolites determined a high antioxidant potential, which was revealed in the DPPH and ABTS tests. The free radical scavenging properties of *U. dioica* extract were also confirmed in the in vitro H_2_DCFDA assay, where pretreatment with the extract significantly decreased the ROS levels in H_2_O_2_-treated skin fibroblasts. Bourgeois et al. also described the strong antioxidant properties of *U. dioica* extract in the ferric reducing/antioxidant power (FRAP) and cupric reducing antioxidant capacity (CUPRAC) tests [18]. Furthermore, they found that the extract displays an inhibitory effect on collagenase and elastase [18], which are important enzymes contributing to the degradation of the extracellular matrix in the skin. This results in the development of wrinkles, a decrease in skin firmness, and reduced resilience [35].

The detailed phytochemical investigation revealed a high concentration of caffeic acid derivatives in the extracts, with caffeoylmalic acid and chlorogenic acids (CAs) being the most abundant. These compounds are attributed to significant antioxidant potential, as evidenced by numerous research studies [25,36,37,38]. They exhibited strong effectiveness in both chemical-based and cell-based models, and their mechanism of action involves the direct reduction of ROS and an impact on the cellular antioxidant enzymes responsible for maintaining redox balance in the cells [37].

A protective effect against H_2_O_2_-induced cytotoxicity is another beneficial effect of *U. dioica* extract observed in our investigation. Furthermore, the extract was found to be non-cytotoxic, and it even exhibited a mild proliferative effect. This positive impact on skin fibroblasts may be linked to the presence of caffeic acid (CA) as previous reports suggested that this compound stimulates the proliferation of dermal fibroblasts [36,39].

In addition to their strong antioxidant and protective potential, caffeic acid derivatives also exhibit other highly desirable biological properties for skincare. They demonstrate anti-inflammatory, antibacterial, and anti-yeast effects, possess antiallergic properties, stimulate collagen production, and provide protection against UVB radiation. [40,41]. All of these mentioned features make *U. dioica* extract a valuable cosmetic additive candidate. While there are various types of commercially available nettle-based products, including facial tonics, creams, and shampoos, future plans should involve the development of efficient formulations, such as hydrogels. These matrices have the potential to enhance the effective delivery of phytochemicals into the skin. Furthermore, the study of other parts of the plant, such as the flowers, could be considered with regard to their biological activity and potential in the field of skincare.

## 5. Conclusions

This work aimed to determine the antioxidant and protective properties of U. dioica extract against H_2_O_2_-induced oxidative stress in human skin fibroblasts. The obtained results demonstrate that the extract is rich in phenolic compounds, most of which are caffeic acid derivatives, and that it exhibits a high capacity to scavenge ROS. Furthermore, it positively affects cell viability and metabolic activity and protect cells against H_2_O_2_-induced cytotoxicity. These results suggest that *U. dioica* leaf extract can be considered a highly valuable cosmetic additive.

## Figures and Tables

**Table 1:** Mass data of identified compounds in the extracts from *Urtica dioica*.

No	R_T_ (min.)	Mass Data (m/z-H)	Fragment (m/z-H)	Formula	Δ ppm	Component
**1**	1.53	191.05657	-	C_7_H_12_O_6_	2.39	Quinic acid *
**2**	3.93	341.08797	(135, 179)	C_15_H_18_O_9_	0.48	Caffeoylhexoside
**3**	4.79	315.07261	(152)	C_13_H_16_O_9_	1.44	Dihydroxybenzoic acid hexoside
**4**	6.28	371.06167	(209, 135, 179)	C_15_H_16_O_11_	−0.85	Caffeoylglucaric acid
**5**	8.29	447.11421	(209, 135, 179)	C_18_H_24_O_13_	−0.46	Caffeic derivative
**6**	8.34	353.08872	(191, 135, 179)	C_16_H_18_O_9_	2.58	Neochlorogenic acid *
**7**	13.57	353.08887	(191, 135, 179)	C_16_H_18_O_9_	3.01	Chlorogenic acid *
**8**	14.25	353.08863	(191, 135, 179)	C_16_H_18_O_9_	2.33	Cryptochlorogenic acid *
**9**	15.01	179.03551	(135)	C_9_H_8_O_4_	2.93	Caffeic acid *
**10**	15.67	295.04614	(135, 179)	C_13_H_12_O_8_	0.67	Caffeoylmalic acid
**11**	17.21	337.09191	(191, 163, 173)	C_16_H_18_O_8_	−2.90	p-Coumaroylquinic acid (I)
**12**	19.15	337.09253	(191, 163)	C_16_H_18_O_8_	−1.07	p-Coumaroylquinic acid (II)
**13**	21.65	193.05011		C_10_H_10_O_4_	−2.67	Ferulic acid *
**14**	28.57	609.14562	(300, 463)	C_27_H_30_O_16_	−0.80	Rutin *
**15**	29.09	463.08785	(300)	C_21_H_20_O_12_	−0.75	Isoquercetin *
**16**	32.03	505.09948	(300)	C_23_H_22_O_13_	1.41	Quercetin derivative
**17**	34.40	593.14999	(285)	C_27_H_30_O_15_	−2.03	Kaempferol rutinoside *
**18**	36.30	623.16183		C_28_H_32_O_16_	0.11	Unknown flavonoid

* identification was confirmed using standard.

**Table 2:** The results of quantification of the polyphenols (µg/mL ± SD) found in *U. dioica* extracts.

No	Component	DRW	DRE	DRG	DW	DE	DG
**1**	Quinic acid	10.8 ± 0.8	7.8 ± 0.4	6.2 ± 0.4	12.1 ± 0.5	4.9 ± 0.2	6.1 ± 0.3
**3**	Dihydroxybenzoic acid hexoside *	0.65 ± 0.04	0.67 ± 0.05	0.58 ± 0.04	0.53 ± 0.03	0.52 ± 0.03	0.57 ± 0.04
**7**	Chlorogenic acid	64.9 ± 3.1	50.2 ± 3.4	47.3 ± 3.3	nd	nd	nd
**8**	Cryptochlorogenic acid	5.2 ± 0.4	3.4 ± 0.2	4.2 ± 0.3	nd	nd	nd
**9**	Caffeic acid	2.3 ± 0.1	1.6 ± 0.1	1.7 ± 0.1	nd	nd	nd
**10**	Caffeoylmalic acid	114.4 ± 8.7	90.0 ± 6.1	86.6 ± 5.7	nd	nd	nd
**12**	p-Coumaroylquinic acid **	8.9 ± 0.6	7.7 ± 0.4	6.9 ± 0.4	12.1 ± 0.6	12.1 ± 0.5	12.5 ± 0.5
**13**	Ferulic acid	1.9 ± 0.1	1.5 ± 0.1	1.4 ± 0.1	2.5 ± 0.1	1.9 ± 0.1	2.3 ± 0.2
**14**	Rutin	8.9 ± 0.3	8.4 ± 0.4	8.2 ± 0.3	nd	nd	nd
**15**	Isoquercetin	1.4 ± 0.1	1.2 ± 0.1	1.8 ± 0.1	nd	nd	nd

nd—not detected, *—quantification was based on dihydroxybenzoic acid calibration curve, **—quantification was based on p-coumaric acid calibration curve.

## Data Availability

The data presented in this study are available on request from the corresponding author.

## Supplementary Materials

The following supporting information can be downloaded at: https://www.mdpi.com/article/10.3390/life13112182/s1, Figure S1: Chromatogram of *Urtica dioica* extract registered at wavelength of 320 nm, Figure S2: BPC chromatograms of extracts obtained through direct extraction with 5% ethanol in water (red line), 10% polypropylene glycol in water (green line), and water (blue line), Figure S3: BPC chromatograms of extracts obtained through the re-dissolution of a methanol/water extract in water (red line), 5% ethanol in water (green line), and 10% polypropylene glycol in water (blue line), Table S1: Parameters used for quantification of components from *Urtica dioica*, Table S2: Mass spectra and UV-Vis spectra of main component identified in *Urtica dioica* extract. The list of compounds is included in Table 1.
