# Supplementary material for: Protective Effect of Urtica dioica Extract against Oxidative Stress in Human Skin Fibroblasts
Source: Life (Basel). 2023 Nov 9;13(11):2182. doi: 10.3390/life13112182 (PMC10672499; doi:10.3390/life13112182)
Supplement: Supplementary file 1 [file life-13-02182-s001.zip › life-2670398-supplementary.pdf]

life

MDPI

Article

Protective Effect of Urtica dioica Extract against Oxidative Stress in Human Skin Fibroblasts

Agnieszka Skalska-Kamińska [1], Weronika Wójciak [1], Magdalena Żuk [1], Roman Paduch [2,3] and Magdalena Wójciak [1,*]

[1] Department of Analytical Chemistry, Medical University of Lublin, Chodźki 4a, 20-093 Lublin, Poland
[2] Department of Virology and Immunology, Institute of Biological Sciences, Faculty of Biology and Biotechnology, Maria Curie-Skłodowska University, 19 Akademicka Street, 20-033 Lublin, Poland
[3] Department of General and Pediatric Ophthalmology, Medical University of Lublin, Chmielna 1, 20-079 Lublin, Poland
* Correspondence: magdalena.wojciak@umlub.pl

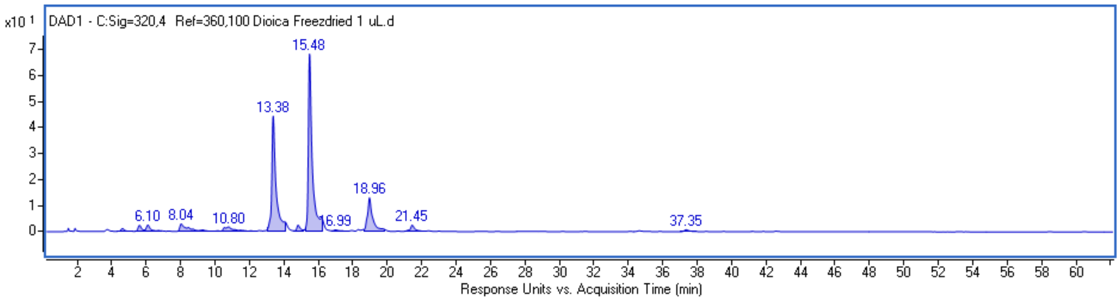

Figure S1. Chromatogram of *Urtica dioica* extract registered at wavelength of 320 nm

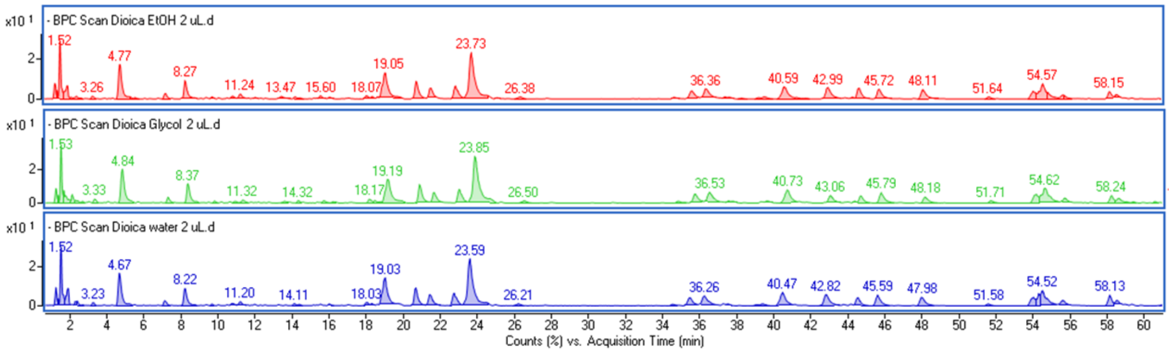

Figure S2. BPC chromatograms of extracts obtained through direct extraction with 5% ethanol in water (red line), 10% polypropylene glycol in water (green line) and water (blue line).

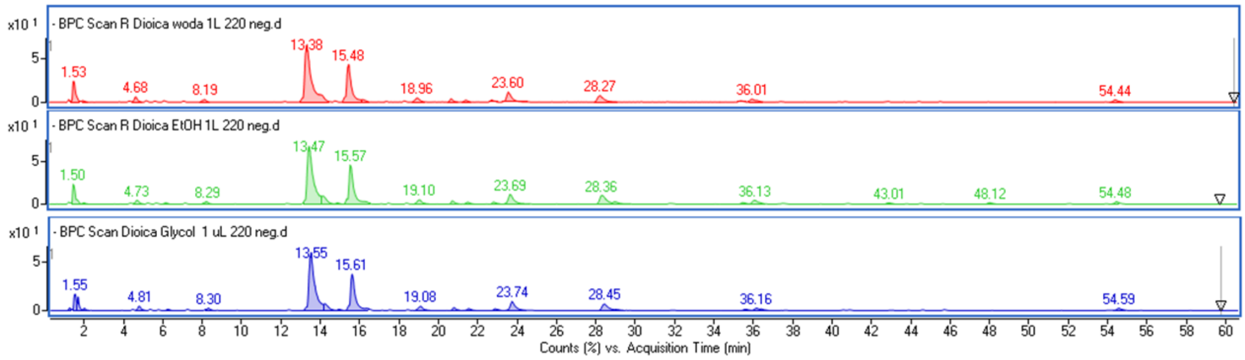

Figure S3. BPC chromatograms of extracts obtained through the re-dissolution of a methanol/water extract in water (red line), 5% ethanol in water (green line) and 10% polypropylene glycol in water (blue line).

Table S1. Parameters used for quantification of components from *Urtica dioica*.

λ (nm)	Component	Range (μg/mL)	Linearity (r^2)	Equation
320	Dihydroxybenzoic acid	0.2-1	0.9997	y = 20.9x + 1.97
326	Chlorogenic acid	10-100	0.9999	y = 49.3x + 5.1
326	Cryptochlorogenic acid	1-10	0.9999	y = 44.3x + 22.8
322	Caffeic acid	1-10	0.9999	y = 119.3x + 15.4
322	Caffeic acid	10-100	0.9998	y = 120.2x – 41.4
310	p-Coumaric acid**	1-10	0.9998	y = 85.7x + 9.7
326	Ferulic acid	0.5-5	0.9998	y = 73.7x + 13.4
350	Rutin	1-10	0.9998	y = 16.4x + 2.3
350	Isoquercetin	0.5-5	0.9997	y = 30.5x + 4.2
**m/z-H**	**Component**	**Range (μg/mL)**	**Linearity (r^2)**	**Equation**
191.055-191.057	Quinic acid	2-20	0.9997	y = 684670x - 64178

Table S2. Mass spectra and UV-Vis spectra of main component identified in *Urtica dioica* extract. The list of compounds is included in Table 1.

	Mass spectrum	UV-Vis spectrum
1	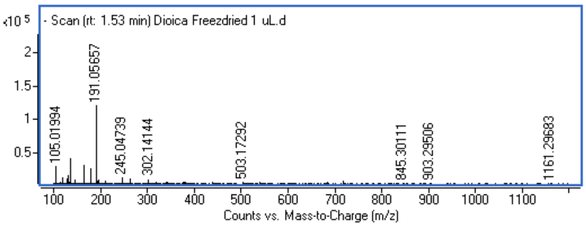	
7	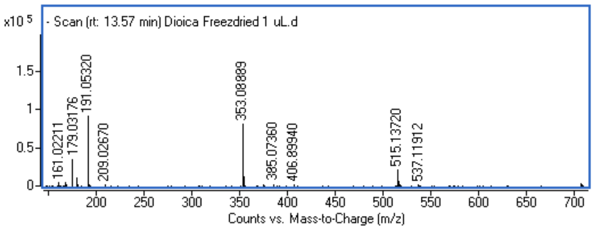	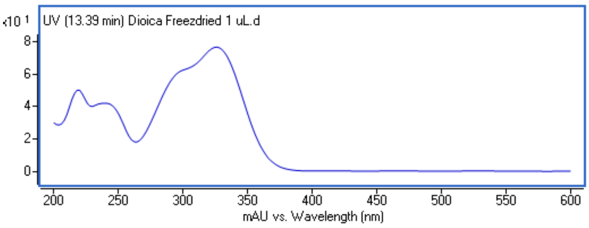
10	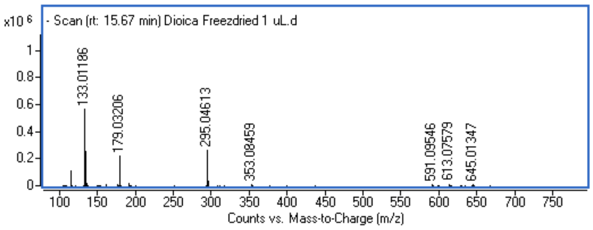	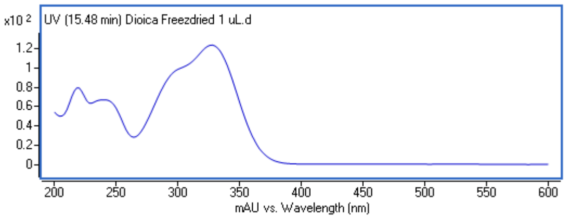
12	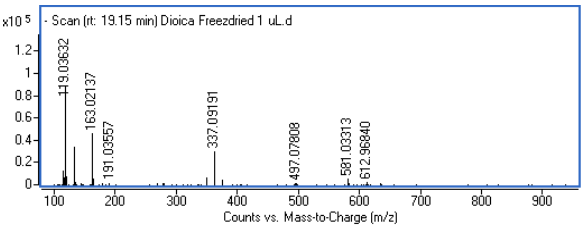	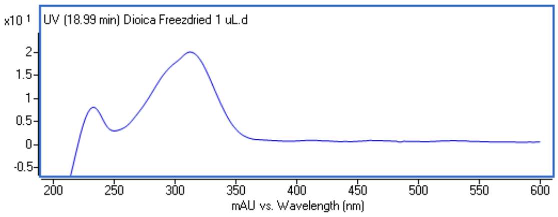
13	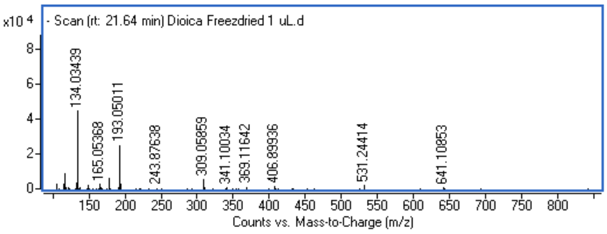	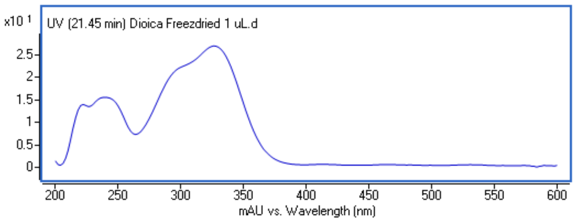
14	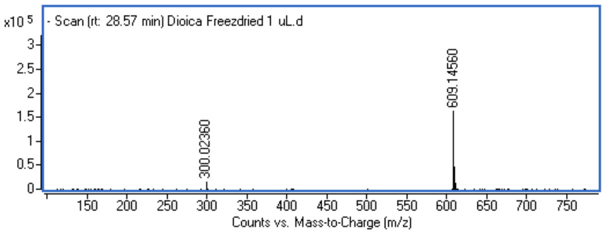	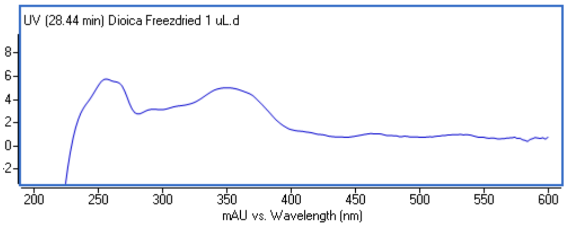

15

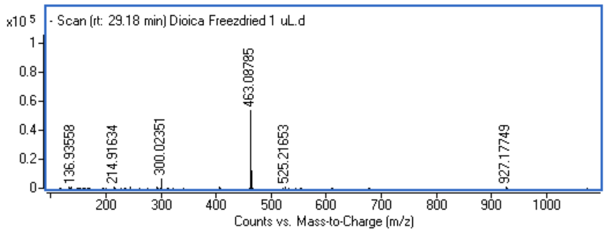
x10 5
- Scan (rt: 29.18 min) Dioica Freezdried 1 uL.d
136.93558
214.91634
300.02351
463.08785
525.21653
927.17749
Counts vs. Mass-to-Charge (m/z)

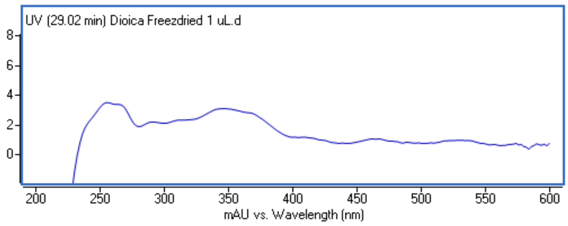
UV (29.02 min) Dioica Freezdried 1 uL.d
mAU vs. Wavelength (nm)
